# Genome-wide scan for selection signatures in six cattle breeds in South Africa

**DOI:** 10.1186/s12711-015-0173-x

**Published:** 2015-11-26

**Authors:** Sithembile O. Makina, Farai C. Muchadeyi, Este van Marle-Köster, Jerry F. Taylor, Mahlako L. Makgahlela, Azwihangwisi Maiwashe

**Affiliations:** Agricultural Research Council-Animal Production Institute, Private Bag X 2, Irene, 0062 South Africa; Department of Animal and Wildlife Sciences, University of Pretoria, Private Bag X 20, Hatfield, 0028 South Africa; Agricultural Research Council-Biotechnology Platform, Private Bag X 5, Onderstepoort, 0110 South Africa; Division of Animal Sciences, University of Missouri, Columbia, MO 65211 USA; Department of Animal, Wildlife and Grassland Sciences, University of Free State, Bloemfontein, 9300 South Africa

## Abstract

**Background:**

The detection of selection signatures in breeds of livestock species can contribute to the identification of regions of the genome that are, or have been, functionally important and, as a consequence, have been targeted by selection.

**Methods:**

This study used two approaches to detect signatures of selection within and between six cattle breeds in South Africa, including Afrikaner (n = 44), Nguni (n = 54), Drakensberger (n = 47), Bonsmara (n = 44), Angus (n = 31) and Holstein (n = 29). The first approach was based on the detection of genomic regions in which haplotypes have been driven towards complete fixation within breeds. The second approach identified regions of the genome that had very different allele frequencies between populations (*F*_ST_).

**Results and discussion:**

Forty-seven candidate genomic regions were identified as harbouring putative signatures of selection using both methods. Twelve of these candidate selected regions were shared among the breeds and ten were validated by previous studies. Thirty-three of these regions were successfully annotated and candidate genes were identified. Among these genes the keratin genes (*KRT222*, *KRT24*, *KRT25*, *KRT26*, and *KRT27*) and one heat shock protein gene (*HSPB9)* on chromosome 19 between 42,896,570 and 42,897,840 bp were detected for the Nguni breed. These genes were previously associated with adaptation to tropical environments in Zebu cattle. In addition, a number of candidate genes associated with the nervous system (*WNT5B*, *FMOD*, *PRELP*, and *ATP2B*), immune response (*CYM*, *CDC6*, and *CDK10*), production (*MTPN*, *IGFBP4*, *TGFB1*, and *AJAP1*) and reproductive performance (*ADIPOR2*, *OVOS2*, and *RBBP8*) were also detected as being under selection.

**Conclusions:**

The results presented here provide a foundation for detecting mutations that underlie genetic variation of traits that have economic importance for cattle breeds in South Africa.

**Electronic supplementary material:**

The online version of this article (doi:10.1186/s12711-015-0173-x) contains supplementary material, which is available to authorized users.

## Background

South Africa has a rich variety of cattle breeds, i.e. *Sanga* types (e.g. Afrikaner and Nguni), European *Bos taurus* breeds (e.g. Angus, Hereford and Holstein), those of unclear origin such as the Drakensberger breed, and some locally developed composite breeds (e.g. Bonsmara and Brangus). Nguni and Afrikaner cattle are indigenous breeds that have been farmed for centuries in South Africa [1]. During the mid-20th century, Afrikaner cattle were crossbred with *Bos taurus* breeds that originated from Europe such as Hereford and Shorthorn to develop the Bonsmara breed [1]. Afrikaner, Drakensberger and Bonsmara cattle are used for beef production, while the Nguni is a dual-purpose breed that is farmed for beef and milk production, particularly in traditional farming systems. Afrikaner cattle are well adapted to the veld conditions of the warm, arid and extensive grazing areas of South Africa, and are known to have a lower susceptibility to most of the country’s endemic diseases such as redwater, heartwater and gallsickness [2]. Nguni cattle are farmed in a variety of biomes in South Africa, which are characterized by periodic drought, seasonal dry periods and nutritional shortages in the natural veld, and this breed is also resistant to a variety of external and internal parasites and stock diseases [2]. Drakensberger cattle are concentrated in the sourveld regions of South Africa, and are used in extensive and intensive beef production systems. All these breeds have participated in animal recording systems since the early 1960s [3] and have been subjected to selection for traits of economic importance such as reproduction and growth. The process of domestication, subsequent breed formation and artificial selection, coupled with the recent rapid decrease in effective population size from a very large ancestral population, has left detectable signatures of selection in numerous regions of the cattle genome [4]. When selection acts on a mutation, it also affects linked sites and leaves a signature in the flanking chromosomal regions. Signals that can be observed on selected genes include: (1) a spectrum of allele frequencies among closely linked sites that is shifted towards extreme frequencies, (2) an excess of homozygous genotypes, and (3) a high frequency of long haplotypes [5].

The availability of high-density single nucleotide polymorphism (SNP) genotyping assays has made it possible to scan the cattle genome for positions that may have been targeted by selection [6]. The detection of signatures of selection is relevant since it may contribute to better understand the mechanisms that underlie traits that have been exposed to intensive natural and artificial selection. Such information also provides important insights into the mechanisms of evolution [7], selection of loci for breeding and selection programs [8] and is useful for the annotation of significant functional genomic regions [9]. However the detection of selection signatures is challenging for several reasons. First, the effects of selection on the distribution of genetic variation can be confounded with patterns of genetic variation that are caused by demographic events such as the size, structure and mating pattern of a population [10]. Adaptive hitchhiking, population expansion and population reduction (e.g. bottlenecks) can also result in an excess of rare alleles [11]. Second, most studies have been conducted using SNP assays that contain only common SNPs. Thus, the variability and distribution of allele frequencies and the levels of linkage disequilibrium (LD) are all strongly affected by this SNP ascertainment bias [9]. Despite these challenges, the detection of signatures of selection has been the focus of several theoretical (simulated) and empirical (observed) studies [8, 12, 13].

Several methods have been used to detect selection signatures, including those based on LD, spectra of allele frequencies and characteristics of haplotype structures in selected populations [14]. These methods have been used to infer genomic regions that were affected by domestication, breed formation and selection for specific production traits in livestock. In chickens, Rubin et al. [15] detected selective sweep regions that are potentially associated with domestication and the specialization of broiler and layer birds using sequence data. They also found a region that harboured the *TSHR* gene that is associated with metabolic regulation and photoperiod control of reproduction in vertebrates. In pigs, putative selective sweeps were reported on chromosomes 1 and 3 [16]. In addition, genomic regions that contain the *IGF2*, *PRLR* and *GHR* genes were shown to have been exposed to intensive selection in pigs [17]. Furthermore, genomic regions that are associated with behaviour, immune response and feed efficiency were detected based on *F*_ST_ (fixation index) estimates of divergence in cattle using high-density SNP assays [4]. Using population differentiation (*F*_ST_) and Integrated Haplotype Score approaches, Qanbari et al. [18] identified 236 genomic regions that are potentially under selection in Holstein cattle. Both approaches suggested selection in the vicinity of the *SIGLEC5* gene on *Bos taurus* chromosome (BTA) 18, a region that was shown to include a major quantitative trait locus (QTL) with large effects on productive life and fertility traits in Holstein cattle [18]. Studies based on sequence data do not suffer from SNP ascertainment bias as do studies that are performed using commercially available SNP assays.

The possibility that variants with large effects may underlie the adaptation of South African cattle breeds has prompted investigations on the genetic basis of adaptation to ticks, parasites, drought and diseases [19–21] and of their ability to produce good quality beef [22]. In a study by Makina et al. [23], some signals of admixture and genetic relatedness were detected between the Afrikaner, Nguni, Drakensberger and Bonsmara breeds. Allowing for six ancestral populations revealed that the Nguni breed shares ancestry with the Afrikaner breed, with approximately 8 % of its genome derived from the Afrikaner breed. The Bonsmara breed shares ancestry with both Nguni (3 %) and Afrikaner (5 %) breeds, while the Drakensberger breed shares 5 % of its genome with the Nguni and Bonsmara and only 3 % with the Afrikaner breed. Besides, the indigenous and locally-developed South African cattle breeds and European *Bos taurus* (Angus and Holstein) breeds have been shown to be clearly differentiated [23], which agrees with their separate histories of domestication and long divergence time periods [24]. However, little is known about the genetic variation that underlies traits of economic importance in cattle breeds of South Africa. Consequently, we conducted a genome-wide scan across six South African cattle breeds to identify genomic regions that have been exposed to strong selection during domestication, breed formation and creation of biological types.

## Methods

### Animal samples and quality control

A total of 249 animals representing the Afrikaner (n = 44), Nguni (n = 54), Drakensberger (n = 47), Bonsmara (n = 44), Angus (n = 31) and Holstein (n = 29) breeds were genotyped using the Illumina BovineSNP50 BeadChip v2 which features 54,609 SNPs distributed throughout the bovine genome with an average spacing of 47 kb [25]. The genotyped samples were derived from a previous study [23] and were approved for this research by the University of Pretoria Ethical Committee (E087-12). Blood, hair and semen were used to extract genomic DNA. These samples were selected based on pedigree data to select against full-sib and half-sib animals in order to maximize the genetic diversity represented within each sampled population. Furthermore, identity-by-descent analysis was performed using the data generated from the Bovine SNP50 BeadChip to select only the individuals with an identity score of less than 0.25 using PLINK version 1.07 [26]. Only SNPs that were uniquely mapped to autosomes on the UMD3.1 assembly were included in the analyses. Samples with more than 10 % missing genotypes were excluded.

Two methods were used for quality control of the data. The first analytical approach detected selective sweeps within each breed by searching for local reductions in genetic variation using minor allele frequencies (MAF). Thus, the BovineSNP50 data were first filtered to retain loci with a call rate per breed of at least 95 % and 51,406 (Afrikaner), 50,870 (Nguni), 50,389 (Drakensberger), 51,242 (Bonsmara), 50,922 (Angus) and 52,294 (Holstein) SNPs remained. The second analytical approach targeted the identification of signatures of divergent selection between breeds using population differentiation (*F*_ST_). Thus, SNPs with a call rate less than 95 % and a MAF less than 2 % across all breeds [26] were removed leaving 45,657 SNPs. Furthermore, SNPs that were in high LD were pruned using *indep 50 5 2* in the PLINK version 1.07 [26]. A total of 21,290 SNPs remained after pruning and were used for the detection of signatures of selection using *F*_ST_. Pruning of SNPs that are in high LD has been shown to reduce the mean SNP heterozygosity within the European cattle breeds that were used to discover the common SNPs for the design of the BovineSNP50 assay and therefore it partially counters the effects of SNP ascertainment bias [27].

### Identification of selection signatures

Combining alternative approaches to detect selection signatures has been suggested as a means of increasing the reliability of these studies [5]. Thus, two methods were used to detect putative selection signatures. The first method searched for strong recent selection signatures, for which haplotypes have been driven to complete fixation within each breed [13]. This is based on the observation that intensive selection for variants ultimately leads to a complete loss of variation within the chromosomal region that surrounds the selected variant and results in the complete fixation of the haplotype that harbours the selected variant [13]. The second method searched for loci with exceptionally high *F*_ST_ owing to differential selection histories between populations, which leads to distortions in allele frequencies between populations at loci that flank the selected variants [12]. This approach is based on the fact that local positive selection tends to reduce the heterozygosity of specific loci in a population by increasing the frequency of one allele in one breed, which results in a higher proportion of between-breed than within-breed genetic variation [10].

To identify signatures of intensive recent selection within South African cattle breeds, the BovineSNP50 data were analysed separately for each breed taking into consideration that the total number of variable SNPs differed between breeds because of the ascertainment bias due to how SNP discovery is performed for the design of the BovineSNP50 assay [13]. To identify selective sweeps within each breed, a minimum number of five breed-specific contiguous monomorphic SNPs (Table 1) spanning 100 kb (UMD3.1 coordinates) and with a MAF lower than 0.01 was required. To allow for the possibility of new mutations, genotyping errors and assembly errors, which may have incorrectly assigned a SNP to a sweep, a minimum MAF of ≤0.01 was allowed [13].

**Table 1:** Number of animals genotyped from six breeds

Breed	Breed type	Primary historical use	Contiguous bovineSNP50 loci^a^	Number of monomorphic SNP50 loci	Number of individuals genotyped
Afrikaner	Sanga	Beef	8	15,791	42
Nguni	Sanga	Beef/milk	7	10,059	54
Drakensberger	Sanga	Beef	5	6543	47
Bonsmara	Composite	Beef	6	8278	44
Angus	*Bos taurus*	Beef	6	6861	31
Holstein	*Bos taurus*	Milk	6	8463	29

^a^Number of contiguous loci that span at least 100 kb and have a minor allele frequency ≤0.01 required to declare a selective sweep for each breed

To determine the appropriate number of contiguous SNPs within each breed with a MAF ≤0.01 to declare a selective sweep, a trade-off between type 1 error and the size of the detected signature was required. According to Ramey et al. [13], if 15 % of the SNPs are monomorphic within a breed (Table 1), the probability that N contiguous SNPs are monomorphic is 0.15 N under the null hypothesis of no selective sweep in the genome. For example, assuming independence, and testing of 51,406 (Afrikaner), 50,870 (Nguni), 50,389 (Drakensberger), 51,242 (Bonsmara), 50,922 (Angus) and 52,294 (Holstein) SNPs on 29 autosomes, we would expect to find 0.15 N × (52,294-29 × (N − 1)) regions where N contiguous SNPs have fixed alleles. For N = 5, this corresponds to 4.0 false positives per breed but only 0.6 false positives when N = 6. While increasing the number of contiguous monomorphic SNPs decreases the number of type 1 errors, it also increases the size of the signature that can be detected to, on average, (N − 1) × 47 kb [13]. Therefore, an intermediate balance of these conflicting constraints was chosen (Table 1) based on the idea that signatures identified in two or more breeds or any sweep that overlaps with previously reported sweeps would provide strong evidence for the existence of the sweep and these should share a common haplotype.

To identify genomic regions that have been subjected to local positive selection among South African cattle breeds, we identified regions of the genome that showed high levels of population subdivision between the breeds [10, 28] using population-specific *F*_ST_ [29]. Unbiased estimates of *F*_ST_ as described by Weir and Cockerham [29] were calculated using SNP Variation Suite (SVS) version 8 [30] for each of the SNPs between all (15) pairs of cattle breeds in this study. Values were interpreted using the qualitative guidelines proposed by Wright [31] where an *F*_*ST*_ greater than 0.25 indicates very great differentiation, *F*_ST_ ranging from 0.15 to 0.25 great differentiation, from 0.05 to 0.15 moderate differentiation and an *F*_ST_ less than 0.05 little differentiation among the populations.

Unbiased estimates of *F*_ST_ can assume negative values, which do not have a biological interpretation, thus all negative values were set to 0.0 [29]. To determine the variation in allele frequency between loci, an empirical genome distribution of *F*_ST_ values for all autosomal SNPs was constructed across the breeds.

Based on the relationships between breed pairs, the most differentiated breed pairs were selected as candidate pairs for the detection of signatures of selection. Thus, the dairy Holstein was used as the control breed for the analyses on the other five beef breeds, while the Angus beef breed (British origin and less adapted to tropical regions) was used for all four tropically-adapted South African beef breeds to search for signatures of selection that may be associated with environmental adaptation.

A sliding window of five SNPs was used to compute averages for *F*_ST_ and the resulting smoothed *F*_ST_ values for each of the compared breed pairs were plotted against chromosomal coordinates for the central SNP in the window based on the UMD3.1 assembly using SNP Variation Suite (SVS) version 8.1 (SVS 8.1; Golden Helix Inc., Bozeman, Montana) [30]. The most differentiated regions representing the 2 % SNPs with the highest *F*_ST_ (≥0.25) were identified and these were considered to be under selection.

### Annotation and functional analysis of identified genomic regions

Genomic coordinates for all identified selected regions were used for the annotation of genes that were fully or partially contained within each selected region using the University of California, Santa Cruz Genome Browser [32]. The functions and pathways in which these genes are involved were assessed using Panther [33]. In addition, the Bovine QTL database available online at http://www.animalgenome.org/cgi-bin/QTLdb/BT/search was searched to identify any overlap with previously published bovine QTL within the candidate regions.

## Results

### Fixed haplotypes

Descriptive data characteristics such as MAF, percentage of polymorphic SNPs and Hardy–Weinberg equilibrium for the breeds under study were previously reported [23]. Table 2 shows putative selective sweeps detected within each breed, identified by detecting haplotypes that showed complete fixation.

**Table 2:** Potential candidate genes and previously detected QTL within detected selective sweep regions within breeds

Breed^a^	BTA	UMD3.1 coordinate (bp)	Number of SNPs	Size (kb)	Candidate genes	QTL
ANG	1	89,563,554–89,734,339	6	170.79	*KCNMB3, PIK3CA, ZMAT3*	Body length, withers height, hip width
DRA	1	115,420,906–115,619,350	5	198.44	–	Non return rate, calving ease
BON	3	62,887,463–63,196,635	6	309.17	*GNG5, RPF1*	Milk protein percentage, marbling score, dystocia
AFR	4	102,570,116–103,100,577	6	530.46	*MTPN*	Parasites, marbling score, fat thickness,
DRA	5	28,859,701–29,043,711	5	184.01	*HOXC12, HOXC13*	Udder height, intramuscular fat, milk yield, longissimus muscle area
DRA & BON	5	109,333,059–109,478,057	6	145.00	*WC1, WC1.3*	Calving ease, milk fat, ovulation rate, milk yield, marbling score
BON	6	102,546,791–102,779,196	8	232.41	*ZNF280B,NUCB2, KBTBD1*	Interval to first oestrus after calving, marbling score
HOL	7	63,608,866–63,778,905	6	170.04	*ATOX1, G3BP1, GLRA1*	Somatic cell count, milking speed, tick resistance, heel depth, feed conversion ratio
	7	72,882,903–73,126,315	8	243.41	–	Somatic cell count, milking speed
BON	8	24,844,168–25,057,606	6	213.44	*KIAA1797*	First service conception rate, fat thickness, body weight, somatic cell and marbling score
AFR	10	40,135,969–40,460,414	5	324.45	–	Milk protein yield, milk fat, strength and body weight
ANG & HOL	10	70,871,943–71,022,679	7	150.74	*OTX2*	Milk protein yield, teat length, tick resistance, social separation walking and running
HOL	13	12,076,103–12,276,846	6	200.74	–	Body weight, somatic cell count, teat placement
	13	15,456,721–15,683,571	6	226.85	–	Body weight, somatic cell count, teat placement, udder depth
NGU	13	78,430,096–78,793,099	8	363.00	*KCNB1, PTGIS*	Residual feed intake, body weight (slaughter), weaning weight, teat length
ANG & HOL	16	45,425,579–45,874,144	7	448.57	*AJAP1*	Abomasum displacement, residual feed intake, carcass weight, bone percentage, calving ease
BON	16	51,195,450–51,357,613	6	162.16	*PDPN*	Abomasum displacement, residual feed intake, carcass weight, body weight (weaning and birth), calving ease
HOL	18	44,880,710–45,044,333	6	163.62	*DNAH2, TMEM88, GUCY2D*	Calf size, subcutaneous fat thickness, gastrointestinal nematode burden, residual feed intake, somatic cell
HOL	19	27,734,700–28,060,683	9	325.98	*ALOX15B* *ALOX12B*	Calf size, residual feed intake, milk fat yield
BON	24	34,248,516–34,415,701	6	167.19	*RBBP8*	Stillbirth, udder depth, interval to first oestrus after calving, oleic acid content, weaning weight, somatic cell height

^a^Breed abbreviations and names: *AFR* Afrikaner, *NGU* Nguni, *DRA* Drakensberger, *BON* Bonsmara, *ANG* Angus and *HOL* Holstein

**Fig. 1:**
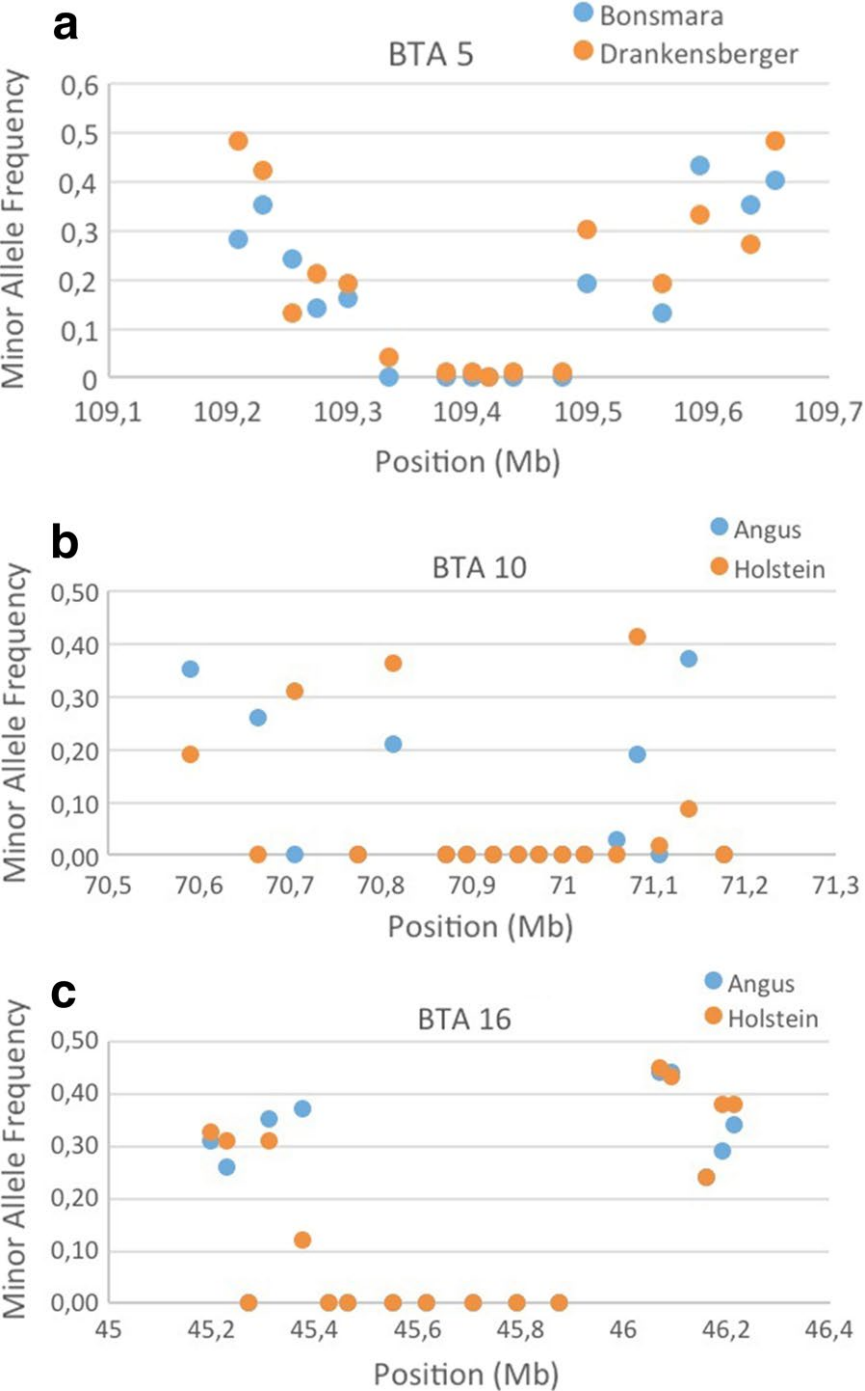
Selective sweep regions shared between two breeds. **a** Bonsmara and Drakensberger. **b** Angus and Holstein. **c** Angus and Holstein

Twenty candidate genomic regions on 13 chromosomes were identified as harbouring putative selective sweeps (Table 2). Putative signatures of selection were identified for all six breeds i.e. ranging from one region (Nguni) to six regions (Holstein) per breed. Seventeen predicted putative signatures were breed-specific and three were shared between breeds with one shared between Drakensberger and Bonsmara (BTA5) and two between Angus and Holstein (BTA10 and 16) (Fig. 1). The average size of the breed-specific sweeps was 267.54 kb, ranging from 162.16 to 530.46 kb while the average size for the common signatures was 245.86 kb, ranging from 95.94 to 448.56 kb. No common sweeps were found between the Afrikaner, Nguni and Drakensberger breeds using the method for which haplotypes were fixed.

### Highly differentiated genomic regions

**Fig. 2:**
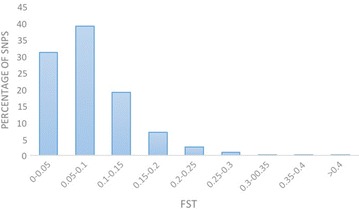
Genome-wide distribution of *F*_ST_ across all autosomes for all 15 breed comparisons

The empirical genome-wide distribution of *F*_ST_ values for all autosomal SNPs was constructed to examine variation in allele frequency between loci (Fig. 2). The distribution was highly skewed towards small *F*_ST_ values. About 31 % of SNPs had an *F*_ST_ less or equal to 0.05 while only 2 % had an *F*_ST_ greater or equal to 0.25. This was consistent with other studies [28, 34, 35] that observed a skewed *F*_ST_ distribution and agrees with the theory of selection on traits that are primarily governed by many loci of small effect [10].

Using the population differentiation approach, 27 candidate genomic regions were identified as potentially under divergent selection. These regions were distributed across 14 chromosomes (Table 3) indicating that about 8.5 Mb of the sequence in these South African cattle breeds is under strong divergent selection. The average size of the candidate genomic regions under selection was 328.88 kb, with the largest region observed between the Afrikaner and Holstein breeds on BTA16 (860.14 kb) between 73,143 and 933,282 bp and the smallest region observed between the Bonsmara-Holstein pair on BTA20 (85.52 kb) between 11,932,262 and 12,017,779 bp.

**Table 3:** Genomic regions identified as being under divergent selection in six cattle breeds in South Africa and their associated QTL

Breeds^a^	BTA	UMD3.1 coord. (bp)	Nb^b^ of SNPs	Size (kb)	Smoothed*F*_*ST*_	Candidate genes	QTL
AFR vs. HOL, NGU vs. HOL	3	35,255,950–35,785,053	5	529.10	0.28	*KCNA2, CYM PROK1, PROK1, LAMTOR, SLC16A4, UBL4B*	Milk fat percentage, milk protein percentage, body weight, height, somatic cell count
AFR vs. HOL	3	121,025,205–121,374,825	4	349.62	0.44	–	Shear force, fat thickness at the 12th rib
BON vs. HOL	3	99,004,471–99,111,024	3	106.55	0.39	*SCP2, SLCA17,* *NDUFA12, SEMA4A*	Calf size, carcass weight, clinical tick-resistance mastitis, marbling score, calving index
DRA vs. HOL	3	7,957,960–8,391,057	3	433.10	0.25	*NOS1AP*	Non return rate, body weight, longissimus muscle area, milk protein percentage, marbling score
AFR vs. HOL, NGU vs. HOL	5	4,472,786–4,598,476	4	125.69	0.42	–	Tenderness score, teat placement, shear force
AFR vs. HOL, NGU *vs.* HOL	5	114,085,555–114,594,935	3	509.38	0.48	*ERC1, FBXL14, WNT5B, ADIPOR2*	Hip height, rump length, calving ease, height, ovulation, type, rump angle
BON vs. HOL	5	107,242,527–107,451,881	4	209.35	0.36	*OVOS2*	Ovulation rate, calving ease, marbling score, height, milk yield, milk fat
NGU vs. HOL	7	71,038,040–71,240,079	4	202.04	0.25	*EBF1*	Somatic cell count, milking speed, tick resistance, heel depth, social separation–vocalisation
DRA vs. HOL	7	46,109,256–46,700,828	4	591.57	0.34	*CXCL14, SLC25A48, FBXL21, LECT2, TGFBI*	Stillbirth, milking speed, body weight, parasites, milk beta-casein percentage
AFR vs. HOL, DRA vs. HOL	9	105,263,583–105,587,941	3	324.36	0.50	*SFT2D1, BRP44L, RPS6KA2*	Chest depth, scrotal circumference, milk yield, milk alpha-casein percentage, milk protein yield
BON *vs.* HOL, NGU vs HOL	9	15,767,136–15,991,964	3	224.83	0.37	*MYO6, IMPG1*	Clinical mastitis, weaning weight, longissimus muscle area, residual feed intake, milk fat yield
NGU vs. HOL	9	46,632,366–47,246,008	4	613.64	0.43	*PREP*	Clinical mastitis, marbling score, milk protein yield
DRA *vs.* HOL	14	81,125,493–81,269,892	3	144.40	0.40	–	Stature, body weight carcass weight, behaviour, height
AFR vs. HOL	16	73,143–933,282	8	860.14	0.31	*FMOD, PRELP, OPTC, ATP2B4, LAX1, ZC3H11A, SNRPE, REN, TMEM51*	Milk protein yield, height, carcass weight, length of productive life
BON *vs.* HOL, NGU vs. HOL	16	49,386,191–49,867,758	3	481.57	0.41	*DNAJC16, CASP9, CELA2A, CTRC, EFHD2, TMEM51*	Abomasum displacement, milk, carcass weight, calving ease, bone percentage
AFR vs. HOL	18	1,094,150–1,422,084	5	327.93	0.47	*DDX19A, DDX19B, AARS, EXOSC6, MRCL, PDPR, GLG1*	Weaning weight-maternal milk
DRA vs. HOL, BON vs. HOL	18	1,212,743–1,486,363	4	273.62	0.35	*PDPR, GLG1*	Weaning weight-maternal milk
NGU vs. HOL	18	14,757,060–14,758,700	3	487.73	0.28	*CHMP1A, SPATA2, CDK10, FANCA, SPIRE2, TCF25, MC1R*	Dystocia, somatic cell score, longissimus muscle area, fat thickness at the 12^th^ rib, carcass weight, stillbirth, skin pigmentation
NGU vs. HOL	19	42,896,570–42,897,840	4	478.76	0.32	HSPB9, *WIPF2, CDC6, RARA, IGFBP4, TNS4, CCR7, SMARCE1*, K *KRT222, KRT24*-*27*	Intramuscular fat, average daily milk yield, milk capric acid percentage, lauric acid, myristic acid, milk c14 index, hair development
AFR vs. HOL	20	11,932,262–12,017,779	3	85.52	0.41	–	Body weight, average daily gain, longissimus muscle area, somatic cell score
AFR vs. HOL, NGU vs. HOL	21	43,246,618–43,399,424	4	152.81	0.30	–	Somatic cell score, calving ease, carcass weight
DRA vs. HOL	21	59,640,020–59,787,612	3	147.59	0.26	*SERPINA3*-*8*	Calving ease, gastrointestinal nematode burden, weaning weight, body weight (birth), height (mature and yearling)
ANG vs. HOL	22	32,930,704–33,076,318	3	145.61	0.28	*FRMD4B*	Non return rate, calf size, somatic cell
NGU vs. HOL	23	2,019,985–2,247,046	3	227.06	0.34	–	Milk protein yield, height, carcass weight, percentage live sperm after thawing
ANG vs. HOL	23	49,809,003–49,945,187	4	136.18	0.46	–	Body weight, dry matter intake
AFR vs. ANG, NGU vs. ANG, DRA vs. ANG, BON *vs. ANG*	24	54,588,817–54,593,951		393.07	0.45, 0.29, 0.25, 0.25	*DCC*	Gastrointestinal nematode burden, body weight, calving ease, udder attachment, feed conversion ratio, body weight
AFR vs. HOL	27	35,734,689–36,117,365	4	382.68	0.26	–	Dystocia, marbling score, clinical mastitis

^a^Breed abbreviations and names: *AFR* Afrikaner, *NGU* Nguni, *DRA* Drakensberger, *BON* Bonsmara, *ANG* Angus and *HOL* Holstein

^b^ *Nb* Number

**Fig. 3:**
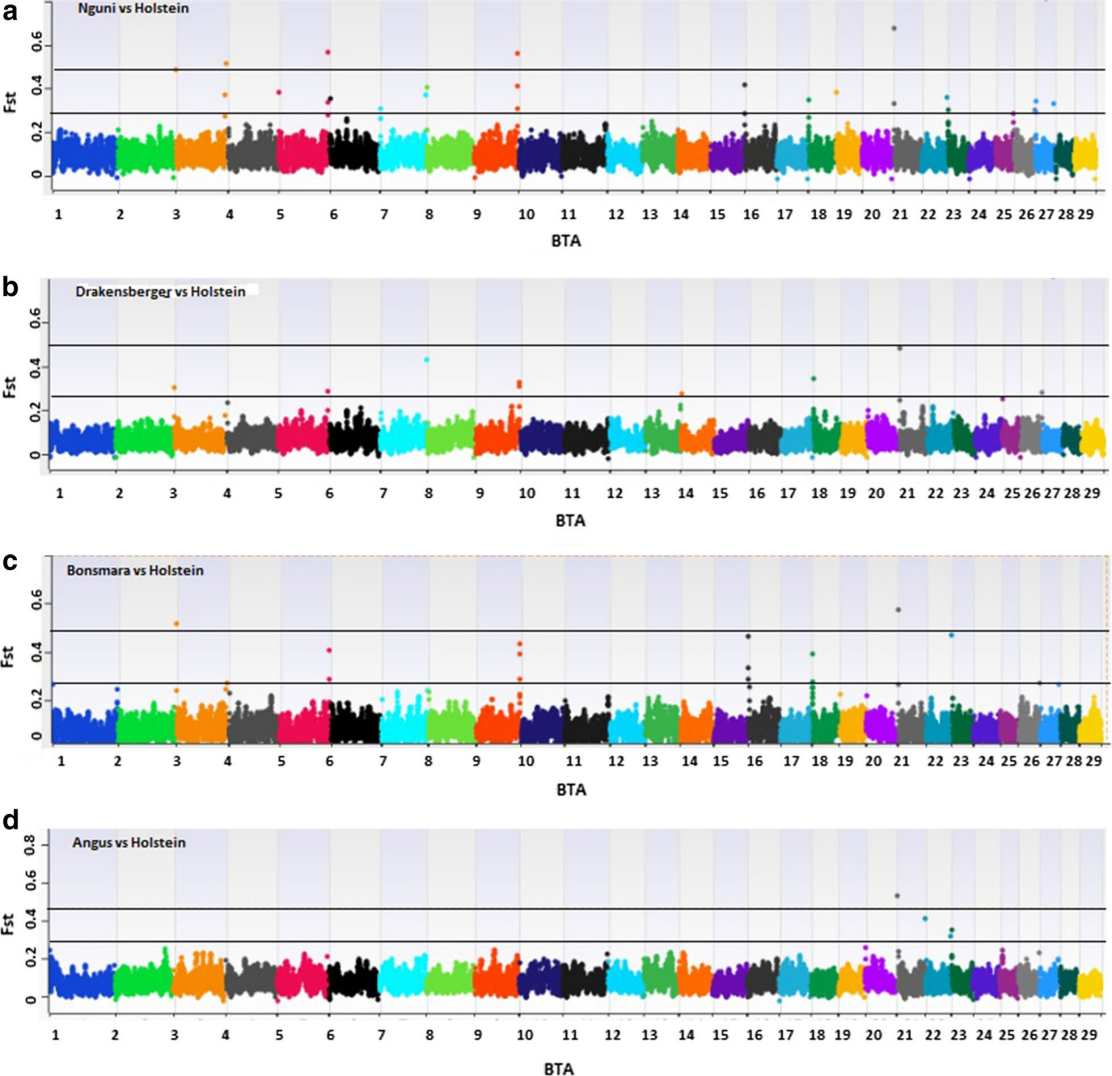
Smoothed *F*_ST_ values for the four breed pair comparisons across the autosomal genome. **a** Nguni vs Holstein. **b** Drakensberger vs Holstein. **c** Bonsmara vs Holstein. **d** Angus vs holstein

**Fig. 4:**
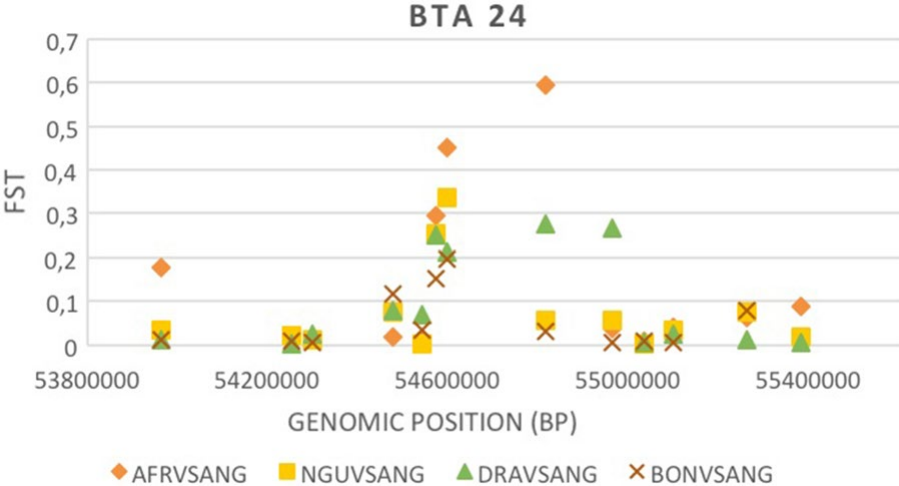
Distribution of *F*_ST_ values for four breed pair comparisons on BTA24. *AFR* Afrikaner, *NGU* Nguni, *DRA* Drakensberger, *BON* Bonsmara and *ANG* Angus

Figure 3 shows Manhattan plots of *F*_ST_ values for the comparisons between the five breeds that generated the largest number of differentiated regions. The number of *F*_ST_ peaks per chromosome varied from 0 to 2 across these comparisons. Nine of these differentiated regions (BTA3, 5, 9, 16, 18, 21 and 24) were shared among breed pairs, with the Afrikaner vs. Holstein and Nguni vs. Holstein pairs sharing the most differentiated regions. The Afrikaner vs. Holstein pair had the largest number of differentiated regions (8) while the Angus vs. Holstein pair had the smallest number (2). The most strongly differentiated region was observed between the Afrikaner and Holstein breeds on BTA9 between 105,263,583 and 105,587,941 bp. Comparisons of Angus vs. Afrikaner, Nguni, Drakensberger and Bonsmara revealed a differentiated genomic region on BTA24 between 54,571,696 and 54,964,769 bp (Fig. 4), which was shared by all of the South African cattle breeds.

### Functional annotation of genomic regions showing evidence of selection

Using the candidate genomic regions that were obtained from both the within- and between-breed analyses, 33 reference sequences were annotated to identify potentially expressed genes. Additional file 1: Table S1 provides full names for all annotated genes in this study. The number of candidate genes obtained per reference sequence varied from one to eight across the genomic regions. Using the Panther [33] website, several candidate genes were linked to important biological functions and pathways in cattle. For example, a region that includes the keratin gene family (*KRT222*, *KRT24*, *KRT25*, *KRT26*, and *KRT27*) and one heat shock protein gene (*HSPB9*) on BTA19 between 42,896,570 and 42,897,840 bp was found to be under selection in Nguni cattle and had previously been associated with tropical adaptation in Zebu cattle [36]. Other regions that included *MTPN* (Afrikaner), *CYM* (Afrikaner and Nguni), *CDC6*, *CDK10*, *EBFI* and *TNS4* (Nguni), *NDUFA12*, *ALOX15B* and *ALOX12B* (Bonsmara) and *SLC25A48* and *SERPINA3*-*8* (Drakensberger) may have been selected due to their association with immune response. Selected regions that contain *ADIPOR2* (Afrikaner), *PTGS* (Nguni), *HOXC12*, *HOXC13*, *WC13* and *OVOS2* (Drakensberger and Bonsmara) may have been selected due to the effects of these genes on reproduction, while those that contain *SLC6A17* and *PREP* may have been selected due to the effects of these genes on fatty acid biosynthesis.

Furthermore, candidate genes related to nervous system development were also identified, for example, *WNT5B*, *FMOD*, *PRELP* (Afrikaner), *CCR7* (Nguni) and *OVOS*, *SLC6A17* (Bonsmara) were localized in selected regions. Candidate genes involved in enzyme regulatory activities, e.g., *MYO6*, *RBBP8* (Bonsmara), *CYM*, *LAX1* (Afrikaner), *ATP2B* (Nguni) and *SLC16A4* (Drakensberger) and genes involved in growth and metabolic processes, e.g., *DDX19A* (Afrikaner), *KCNB1*, *IGFBP* (Nguni), *TGFB1 (*Drakensberger), *MYO6* (Bonsmara), *AJAPI* (Angus) and *ATOX1* (Holstein) were also identified within selected regions. Candidate genes involved in muscle organ development and skeletal development including *KIAAI1797*, *EFHD2* (Bonsmara) and *MTPN*, *TMEM51* (Afrikaner) were also identified as being in regions under selection. Finally, *MC1R* on BTA18 (between 14,757,060 and 14,758,700 bp) which has previously been associated with coat colour in cattle [37] was detected as being under selection in Nguni cattle.

All genomic regions that showed evidence of selection were further analysed to determine whether any of these overlapped with previously reported QTL in cattle. The online database of published bovine QTL revealed that most of the genomic regions overlapped with previously reported regions harbouring QTL that affect milk, fat, carcass, body weight, stature, clinical mastitis, calving ease, tick resistance, gastrointestinal nematode burden and reproductive traits (Tables 2, 3). For example, a region on BTA24 that was detected for the Afrikaner, Nguni, Drakensberger and Bonsmara breeds overlapped with a QTL region that was previously associated with gastrointestinal nematode burden.

The putative signatures of selection that were identified in this study were compared to previously detected bovine sweeps (Table 4). Ten of these candidate genomic regions were supported by previously published data on signatures of selection and clearly harbour variants of large phenotypic effect in cattle.

**Table 4:** Overlapping regions possessing signatures of selection detected in previous studies in cattle

BTA	Position (bp)	Breed	References
1	89,563,554–89,734,339	Angus	[36]
3	99,004,471–99,111,024	Bonsmara	[36, 42]
3	121,025,205–121,374,825	Afrikaner	[44]
5	109,333,059–109,478,057	Bonsmara &Drakensberger	[36, 42]
7	72,882,903–73,126,315	Holstein	[13]
13	15,456,721–15,683,571	Holstein	[13]
16	45,425,579–45,874,144	Angus and Holstein	[13, 36, 42, 43]
16	51,195,450–51,357,613	Bonsmara	[42, 43]
22	32,930,704–33,076,318	Angus	[44]
24	54,588,817–54,593,951	Afrikaner, Nguni, Drakensberger and Bonsmara	[44]

## Discussion

This study used two approaches to identify putative selective sweeps that could be associated with phenotypes, which contribute to domesticability, biological types (adaptation, draught, meat and milk) and to desirable morphologies that might have impacted the extent and distribution of variability within the genomes of South African cattle breeds. The first approach detected complete sweeps that indicate fixation of long haplotypes within breeds as suggested by Ramey et al. [13]. However, the effects of selection on the distribution of genetic variation can be confounded with patterns of genetic variation caused by demographic events such as the size, structure and mating pattern of a population [10]. To distinguish between the effects of selection and those of demographic events, Hayes et al. [38] suggested that the location of the detected loci should be investigated. For instance, demographic events may alter patterns of allele frequencies across the entire genome while selection events are more likely to alter allele frequencies at the loci that are in close vicinity to the mutations that are under selection [38]. In addition, fixed long homozygous haplotypes can also occur due to strong inbreeding following a founder effect [38]; however, a study by Makina et al. [23] demonstrated that the level of inbreeding was relatively low within each of the breeds studied here. Long homozygous haplotypes in breeds that were not included in the design of the BovineSNP50 assay (e.g. Nguni and Afrikaner) could have been created by chance because of the SNP ascertainment bias which would lead to lower overall average MAF for the SNPs on the assay in these breeds. To partially counter this effect, the number of loci required to declare a selective sweep, N, was defined individually for each breed (Table 1) and a larger N was required for breeds with larger numbers of monomorphic and low MAF SNPs.

LD-based methods such as the long range haplotype, extended haplotype homozygosity and integrated haplotype score approaches can be also used to identify genomic regions with unusually long haplotypes that have a high frequency in the population [39]. These approaches are useful to identify variants that have undergone a partial or incomplete selective sweep, in which a new mutation has a frequency that has risen to a modest value in the population but has yet to reach fixation [40]; however these approaches are somewhat sensitive to marker density, which was relatively low in this study. While the across-population extended haplotype homozygosity test can compare haplotype lengths between populations to control for local variation in recombination rate [41], signals of strong recent selection were analyzed within each breed.

The second approach detected genomic regions with high *F*_ST_ between African and European breed pairs using sliding windows throughout the genome [14] to reveal differentiation that could result from different selection histories for production or adaptation to local environments. However, such differentiation could be caused by drift. In contrast to the first approach, the *F*_ST_ approach can detect different types of selection signatures [40], which may explain why the two methods did not produce overlapping signals. One of the limitations associated with the first approach was the calibration relative to the size of the sweeps. While intensive selection in a small population can cause the rapid fixation of a long haplotype, weak selection in a large population would result in the fixation of only a short haplotype, which may not be identified with this approach [13]. Because of the requirement that each of the N contiguous loci should have a MAF less than α, for a small α, N was chosen to be sufficiently large so that the probability of observing N contiguous loci with a MAF less than α by chance alone would be very low and a sufficiently small chromosomal region was defined so that the targeted sweeps would not be smaller than 47 × (N − 1) kb, where 47 kb represents the median interval between SNPs on the BovineSNP50 assay [13]. Furthermore, the design of the BovineSNP50 assay led to lower average MAF and larger numbers of monomorphic SNPs for the Afrikaner and Nguni breeds, which are phylogenetically distant from the breeds that were used to discover the SNPs on the assay [25]. To adjust for this phylogenetic bias, N was individually defined for each breed (Table 1) and a larger N was required for breeds with larger numbers of monomorphic and low MAF SNPs. Finally, the ascertainment bias of common SNPs in the design of the BovineSNP50 assay might explain the inability to detect common sweeps among the Afrikaner, Nguni and Drakensberger breeds using the first analytical method.

Overall, this study detected 47 candidate genomic regions that are potentially either historically or currently under selection within and between six cattle breeds in South Africa. Twenty of these candidate genomic regions were detected within breeds and 27 were detected as regions that had diverged between breeds. In addition, 12 of these candidate genomic regions were shared between breeds and ten had previously been reported [13, 36, 42–44]. Furthermore, no putative selection signatures were predicted to be shared across the South African (indigenous and locally developed) and *Bos taurus* cattle breeds (Angus and Holstein), which is probably due to the different environmental and demographic forces to which these breeds were exposed during breed formation [2].

Domestication has caused considerable changes in the morphology and behaviour of livestock species, as has artificial selection for the specific traits that were selected during breed formation and subsequently for specific breeding objectives [17]. Coat colours are easily identifiable phenotypes that probably played an important role in selection before farmers gained access to objective measurements [17]. In certain breeds, such as Nguni, colour patterns have cultural connotations and coloured hides have different economic values [1]. The melanocyte stimulating hormone receptor gene (*MC1R*) on BTA18 between 14,757,060 and 14,758,700 bp, which influences the production of eumelanin and pheamelanin pigment and is responsible for the pigmentation of skin, eyes and hair [45], was found to be differentially selected between Holstein and Nguni cattle but not between the South African Afrikaner (red), Drakensberger (black) or Bonsmara (red) breeds. This could be due to specific alleles at the *MC1R* gene that are under selection in the Nguni breed. Ramey et al. [13] observed a sweep at *MC1R* in Hanwoo cattle which are yellow. Furthermore, Stella et al. [43] and Flori et al. [46] reported that the *MC1R* gene was under selection in cattle. *MC1R* has been proposed to have three alleles, i.e. *E*^*D*^ for breeds with a black coat (e.g., Holstein, Angus and Murray Grey), *e* for breeds with recessive red coat (e.g., Limousin, Shorthorn and Hereford) and *E*^+^, also called “wild type” for all other breeds except Hereford [47]. The dominant *E*^*D*^ allele is responsible for black coat colour, whereas the recessive *e*/*e* genotype results in red coats. However, wild type *E*^+^*E*^+^ homozygotes may display variable colour patterns, since other genes (e.g., Agouti) can influence the pigments produced [37]. The presence of a putative selection signature on *MC1R* in Nguni cattle, which are characterized by multi-coloured skin patterns that may present various forms (white, brown, golden yellow, black, dappled, or spotted), is of interest and suggests the existence of additional functional alleles at *MC1R* as was also suggested by the presence of a sweep at *MC1R* in yellow Hanwoo cattle [13]. Identifying the mutations that underlie these signals would allow a better understanding of the role of *MC1R* in coat colour patterning in cattle.

Behavioural changes such as reduction in fear and anti-predator responses and increase in sociability are believed to have been selected during domestication [48]. This study detected several putative selection signatures that could be related to the development of the nervous system as well as the regulation of a wide range of tissue and cell functions including behaviour, for example, regions harbouring *WNT5B*, *FMOD*, and *PRELP* (Afrikaner), *CCR7* (Nguni) and *OVOS*, and *SLC6A17* (Bonsmara). The Bovine HapMap Consortium [6] and Gautier et al. [44] also reported selection signatures in regions that contain genes associated with the nervous system of cattle.

South African cattle are farmed in regions that are characterized by periodic drought, seasonal dry periods, and nutritional shortages in the natural veld and are subjected to a variety of external and internal parasites and stock diseases [1]. A number of candidate genes and of gene families that were previously associated with one or more performance attributes of tropical adaptation [36, 44] have been selected in Nguni cattle. For example, keratin genes (*KRT222*, *KRT24*, *KRT25*, *KRT26* and *KRT27*) and one heat shock protein gene (*HSPB9*) on BTA19 between 42,896,570 and 42,897,840 bp were found to be under selection. Heat shock proteins are differentially expressed between indicine and taurine cattle in the tropical environments of Africa and are associated with tropical adaptation in Zebu cattle [36, 44]. Keratins (heteropolymeric structural proteins) form the basis of the structural constituent of the epidermis during epidermal development. Epidermal development occurs in response to adaptation to different climatic and environmental conditions, including tick exposure [49]. In addition, keratins play a role in the formation of the hair shaft [50]. Skin colour and the thickness of the hair directly influence the thermo-tolerance of cattle that live in the tropics [51]. Nguni cattle have a smoother and shinier hair coat than European cattle breeds. Due to these characteristics, Nguni cattle regulate their body temperature and maintain cellular functions more efficiently during heat [20] and also resist better to tick infestation [19]. The absence of such signals in other local cattle breeds such as Afrikaner, Drakensberger and Bonsmara, which also display some ability to survive under extreme conditions [19] may be explained by the fact that the method based on *F*_ST_ is most efficient at detecting differentiation when the region is near fixation for alternate alleles in the breeds compared [39]. Thus, while these loci may be under selection in these breeds, the desirable alleles may still have intermediate frequencies. This agrees with the results of Muchenje et al. [19] and Marufu et al. [21] who reported that Nguni cattle were more resistant to ticks and could better survive to extreme conditions than other local South African breeds.

Several candidate genes that are related to antigen recognition, which is a key process in the development of immune response were identified as being under selection in this study, and include *MTPN (*Afrikaner*)*, *CYM* (Afrikaner and Nguni), *CDC6*, *CDK10*, *KCNBI* and *TNS4* (Nguni), *NDUFA12*, *ALOX15B*, and *ALOX12B* (Bonsmara), and *SLC25A48* and *SERPINA3*-*8* (Drakensberger). The CD family of immune response genes was described by Meissener et al. [52] as being closely involved with molecular functions and pathways of the major histocompatibility complex (MHC). The *TNFAIP8L2* gene has a major role in individual immune homeostasis [53] and the *NDUFA12* gene that has diverging allele frequencies between taurine and Zebu cattle is associated with tick resistance. These observations are consistent with the tolerance of Afrikaner, Nguni, Drakensberger and Bonsmara cattle to various tick and parasitic diseases [19, 21]. Furthermore, candidate genomic regions that include the *MTPN* and *PDPR* (Afrikaner), *DCC* (Afrikaner, Nguni, Drakensberger and Bonsmara), *OTX2* (Angus), *DNAH2*, *TMEM88* and *GUCY2D* (Bonsmara), *EBF1* (Nguni), and *CXCL14* and *SLC25A48* (Drakensberger) genes overlap with previously identified QTL that affect tick resistance and nematode tolerance in cattle.

Several candidate genes within the selected regions are indirectly or directly involved in reproductive pathways including spermatogenesis, ovulation rate, oestrus processes, testis development and prostaglandin development in cattle. These included *OVOS2* (Bonsmara), *ADIPOR2* (Afrikaner and Nguni), *WC1* (Drakensberger and Bonsmara), *RBBP8* (Bonsmara), *SERPINA3*-*8*, *HOXC12* and *HOXC13* (Drakensberger), and *FBXL4* (Afrikaner and Nguni). It has been shown that all these breeds are able to reproduce under harsh environmental conditions; they are considered to be excellent dam lines for crossbreeding, with few calving difficulties [1], which supports the presence of putative selection signatures at loci involved in reproduction that probably occurred during the adaptation of these breeds to South African conditions. In addition, these regions overlap with previously reported QTL associated with reproduction in cattle.

Candidate genes related to growth and muscle development were also detected as being under selection, i.e. *DDX19A*, *TMEM51*, and *MTPN* (Afrikaner), *IGFBP4*, (Nguni), *TGFB1* and *KCNB1*, (Drakensberger), *MYO6*, *KIAAI1797* and *EFHD2* (Bonsmara), *AJAP1* (Angus), and *ATOX1* (Holstein). In addition, some of these regions overlap with previously identified QTL that are associated with stature, body weight and growth in cattle. Furthermore, some of the putative selection signatures detected in this study overlap with previously reported QTL that affect milk yield and quality (BTA3, 5, 10, 16 and 23), feed efficiency (BTA13, 16 and 18), fat thickness (BTA5, 18 and 19), marbling score and carcass weight (BTA3, 5, 16, 20 and 27) as well as somatic cell count (BTA3, 5, 7, 9, 18 and 22).

The overall goal of this study was to identify candidate genomic regions targeted by selection within and between the major cattle breeds of South Africa. The fact that 12 of the identified candidate genomic regions were shared among several of the breeds analysed in this study and that 10 were validated by previous studies reduces the probability of detecting false positives [13]. False positives that could have been introduced by the SNP ascertainment bias or the LD pruning in the *F*_ST_ analyses should be identified in future studies using the BovineHD BeadChip or sequence data. Results of this study provide insights into the genetic mechanisms that underlie traits of economic importance among cattle breeds in South Africa in particular with regard to adaptation to tropical and subtropical environments via increased resistance to tick and parasite-borne diseases and enhanced reproduction and production potential.

## Conclusions

This study represents the first attempt to localize candidate genomic regions targeted by selection in breeds adapted to South African conditions. Several candidate genomic regions either directly or indirectly involved in tropical adaptation, immune response activation, tick and parasite resistance, production and reproduction performance were detected. Moreover, candidate selected regions that overlap with QTL reported in the cattle QTL database provide additional evidence for the significance of the detected regions under selection. This study identified candidate loci that are important for the development of South African cattle breeds and should be prioritized for functional dissection.

## Additional file

10.1186/s12711-015-0173-x Symbols and names for all annotated candidate genes.

## References

[1] Scholtz MM (2010). Beef breeding in South Africa.

[2] Bonsma JC. Cross-breeding, breed creation and the genesis of the Bonsmara. In: Livestock production: A global approach. Cape Town: Tafelberg Publishers Ltd.; 1980. p. 126–36.

[3] Van Marle-Köster E, Webb EC (2014). A perspective on the impact of reproductive technologies on food production in Africa. Current and future reproductive technologies and world food production. Adv Exp Med Biol.

[4] Gibbs RA, Taylor JF, Van Tassell CP, Barendse W, Eversole KA, The Bovine HapMap Consortium (2009). Genome-wide survey of SNP variation uncovers the genetic structure of cattle breeds. Science.

[5] Simianer H, Ma Y, Qanbari S. Statistical problems in livestock population genomics. In Proceedings of the 10th World Congress of Genetics Applied to Livestock Production: 17-22 August 2014; Vancouver. 2014. https://asas.org/docs/default-source/wcgalp-proceedings oral/202_paper_10373_manuscript_1346_0.pdf?sfvrsn=2.

[6] Nielsen R (2005). Molecular signatures of natural selection. Annu Rev Genet.

[7] Otto SP (2000). Detecting the form of selection from DNA sequence data. Trends Genet.

[8] Vitalis R, Dawson K, Boursot P (2001). Interpretation of variation across marker loci as evidence of selection. Genetics.

[9] Nielsen R, Yang Z (1998). Likelihood models for detecting positively selected amino acids sites and applications to the HIV-1 envelope gene. Genetics.

[10] Akey JM, Zhang G, Zhang K, Jin L, Shriver MD (2002). Interrogating a high-density SNP map for signatures of natural selection. Genome Res.

[11] Tajima F (1989). Statistical method for testing the neutral mutation hypothesis by DNA polymorphism. Genetics.

[12] Sabeti PC, Reich DE, Higgins JM, Levine HZ, Richter DJ, Schaffner SF (2002). Detecting recent positive selection in the human genome from haplotype structure. Nature.

[13] Ramey HR, Decker JE, McKay SD, Rolf MM, Schnabel RD, Taylor JF (2013). Detection of selective sweeps in cattle using genome-wide SNP data. BMC Genomics.

[14] Helyar SJ, Hemmer-Hansen J, Bekkevold D, Taylor MI, Ogden R, Limborg MT (2011). Application of SNPs for population genetics of non-model organisms: new opportunities and challenges. Mol Ecol Resour.

[15] Rubin CJ, Zody MC, Eriksson J, Meadows JR, Sherwood E, Webster MT (2010). Whole-genome resequencing reveals loci under selection during chicken domestication. Nature.

[16] Groenen MA, Amaral A, Megens HJ, Larson G, Archibald AL, Muir WN, et al. The porcine HapMap project: Genome-wide assessment of nucleotide diversity, haplotype diversity and footprints of selection in the pig. In: Proceedings of the International Plant and Animal Genome XVIII Conference: 9–13 January 2010; San Diego. W609. 2010. http://comparativegenomics.illinois.edu/publications/abstract/porcine-hapmap-project-genome-wide-assessment-nucleotide-diversity-haplotype.

[17] Andersson L, Georges M (2004). Domestic-animal genomics: deciphering the genetics of complex traits. Nat Rev Genet.

[18] Qanbari S, Gianola D, Hayes B, Schenkel F, Miller S, Moore S (2011). Application of site and haplotype-frequency based approaches for detecting selection signatures in cattle. BMC Genomics.

[19] Muchenje V, Dzama K, Chimonyo M, Strydom PE, Raats JG (2009). Relationship between pre-slaughter stress responsiveness and beef quality in three cattle breeds. Meat Sci.

[20] Muchenje V, Dzama K, Chimonyo M, Raats JG, Strydom PE (2008). Tick susceptibility and its effects on growth performance and carcass characteristics of Nguni, Bonsmara and Angus steers raised on natural pasture. Animal.

[21] Marufu MC, Qokweni L, Chimonyo M, Dzama K (2011). Relationships between tick counts and coat characteristics in Nguni and Bonsmara cattle reared on semiarid rangelands in South Africa. Ticks Tick Borne Dis.

[22] Strydom PE, Naudé RT, Smith MF, Kotzé A, Scholtz MM, van Wyk JB (2001). Relationships between production and product traits in subpopulations of Bonsmara and Nguni cattle. S Afr J Anim Sci.

[23] Makina SO, Muchadeyi FC, van Marle-Köster E, MacNeil MD, Maiwashe A (2014). Genetic diversity and population structure among six cattle breeds in South Africa using a whole genome SNP panel. Front Genet.

[24] McKay SD, Schnabel RD, Murdoch BM, Matukumalli LK, Aerts J, Coppieters W (2008). An assessment of population structure in eight breeds of cattle using a whole genome SNP panel. BMC Genet.

[25] Matukumalli LK, Lawley CT, Schnabel RD, Taylor JF, Allan MF, Heaton MP (2009). Development and characterization of a high density SNP genotyping assay for cattle. PLoS One.

[26] Purcell S, Neale B, Todd-Brown K, Thomas L, Ferreira MAR, Bender D (2007). PLINK: a toolset for whole-genome association and population-based linkage analyses. Am J Hum Genet.

[27] Lopez Herraez D, Bauchet M, Tang K, Theunert C, Pugach I, Li J, et al. Genetic variation and recent positive selection in worldwide human populations: evidence from nearly 1 million SNPs. PLoS One. 2009;4:e7888. 10.1371/journal.pone.0007888 PMC2775638 19924308

[28] Kijas JW, Townley D, Dalrymple BP, Heaton MP, Maddox JF, McGrath A (2009). A genome wide survey of SNP variation reveals the genetic structure of sheep breeds. PLoS One.

[29] Weir BS, Cockerham CC (1984). Estimating F-statistics for the analysis of population structure. Evolution.

[30] Golden Helix Inc. SNP and Variation Suite Manual 2012, Version 8.1 http://www.goldenhelix.com.

[31] Wright S. Evolution and the genetics of populations. Volume 4: Variability within and among natural populations. University of Chicago Press: Chicago; 1978.

[32] Kent WJ, Sugnet CW, Furey TS, Roskin KM, Pringle TH, Zahler AM (2002). The human genome browser at UCSC. Genome Res.

[33] Mi H, Muruganujan A, Thomas PD (2013). PANTHER in 2013: modeling the evolution of gene function, and other gene attributes, in the context of phylogenetic trees. Nucleic Acids Res.

[34] Moradi MH, Nejati-Javaremi A, Moradi-Shahrbabak M, Dodds KG, McEwan JC (2012). Genomic scan of selective sweeps in thin and fat tail sheep breeds for identifying of candidate regions associated with fat deposition. BMC Genet.

[35] Kijas JW, Lenstra JA, Hayes B, Boitard S, Porto-Neto LR, San Critobal M (2012). Genome-wide analysis of the world’s sheep breeds reveals high levels of historic mixture and strong recent selection. PLoS Biol.

[36] Chan EK, Nagaraj SH, Reverter A (2010). The evolution of tropical adaptation: comparing taurine and zebu cattle. Anim Genet.

[37] Chen SY, Huang Y, Zhu Q, Fontanesi L, Yao YG, Liu YP (2009). Sequence characterization of the MC1R gene in Yak (*Poephagus grunniens*) breeds with different coat colors. J Biomed Biotechnol.

[38] Hayes BJ, Chamberlain AJ, Maceachern S, Savin K, McPartlan H, MacLeod I (2009). A genome map of divergent artificial selection between *Bos taurus* dairy and *Bos taurus* beef cattle. Anim Genet.

[39] Vitti JJ, Grossman SR, Sabeti PC (2013). Detecting natural selection in genomic data. Annu Rev Genet.

[40] Tang K, Thornton KR, Stoneking M (2007). A new approach for using genome scans to detect recent positive selection in the genome. PLoS Biol.

[41] Sabeti PC, Varilly P, Fry B, Lohmueller J, Hostetter E, Cotsapas C (2007). Genome-wide detection and characterization of positive selection in human populations. Nature.

[42] Porto-Neto LR, Lee SH, Sonstegard TS, Van Tassell CP, Lee HK, Gibson JP (2014). Genome-wide detection of signatures of selection in Korean Hanwoo cattle. Anim Genet.

[43] Stella A, Ajmone-Marsan P, Lazzari B, Boettcher P (2010). Identification of selection signatures in cattle breeds selected for dairy production. Genetics.

[44] Gautier M, Flori L, Riebler A, Jaffrézic F, Laloë D, Gut I (2009). A whole genome Bayesian scan for adaptive genetic divergence in West African cattle. BMC Genomics.

[45] Seo K, Mohanty TR, Choi T, Hwang I (2007). Biology of epidermal and hair pigmentation in cattle: a mini-review. Vet Dermatol.

[46] Kemper KE, Saxton SJ, Bolormaa S, Hayes BJ, Goddard ME (2014). Selection for complex traits leaves little or no classic signatures of selection. BMC Genomics.

[47] Flori L, Fritz S, Jaffrézic F, Boussaha M, Gut I, Heath S (2009). The genome response to artificial selection: a case study in dairy cattle. PLoS One.

[48] MacHugh DE, Shriver MD, Loftus RT, Cunningham P, Bradley DG (1997). Microsatellite DNA variation and the evolution, domestication and phylogeography of taurine and zebu cattle (*Bos taurus* and *Bos indicus*). Genetics.

[49] Wang YH, Reverter A, Kemp D, McWilliam SM, Ingham A, Davis CA (2007). Gene expression profiling of Hereford Shorthorn cattle following challenge with *Boophilus microplus* tick larvae. Aust J Exp Agric.

[50] Wu DD, Irwin DM, Zhang YP (2008). Molecular evolution of the keratin associated protein gene family in mammals, role in the evolution of mammalian hair. BMC Evol Biol.

[51] Mattioli RC, Pandey VS, Murray M, Fitzpatrick JL (2000). Immunogenetic influences on tick resistance in African cattle with particular reference to trypanotolerant N’Dama (*Bos taurus*) and trypanosusceptible Gobra zebu (*Bos indicus*) cattle. Acta Trop.

[52] Meissner TB, Liu YJ, Lee KH, Biswas A, van Eggermond M, van den Elsen PJ (2012). NLRC5 cooperates with the RFX transcription factor complex to induce MHC Class 1 gene expression. J Immunol.

[53] Zhang L, Shi Y, Wang Y, Zhu F, Wang Q, Ma C (2011). The unique expression profile of human *TIPE2* suggests new functions beyond its role in immune regulation. Mol Immunol.

