# Supplementary material for: Genome-wide scan for selection signatures in six cattle breeds in South Africa
Source: Genet Sel Evol. 2015 Nov 26;47:92. doi: 10.1186/s12711-015-0173-x (PMC4662009; doi:10.1186/s12711-015-0173-x)
Supplement: Supplementary file 1 — 10.1186/s12711-015-0173-x Symbols and names for all annotated candidate genes. [file 12711_2015_173_MOESM1_ESM.docx]

**Additional file 1**

**Table S1 Symbols and names for all annotated candidate genes**

| **Symbol** | **Gene name** | **Symbol** | **Gene name** |  |
| --- | --- | --- | --- | --- |
| *AARS* | *Alanyl-tRNA synthetase* | *SPATA2* | *Spermatogenesis Associated 2* |  |
| *ADIPOR2* | *Adiponectin receptor 2* | *SPIRE2* | *Spire-Type Actin Nucleation Factor* |  |
| *ATP2B4* | *Plasma membrane calcium-transporting ATPase 4* | *TCF25* | *Transcription Factor 25 (Basic Helix-Loop-Helix)* |  |
| *BRP44L* | *Brain protein 44* | *TGFBI* | *Transforming Growth Factor, Beta-Induced, 68kDa* |  |
| *CASP9* | *Caspase 9, Apoptosis-Related Cysteine Peptidase* | *TMEM51* | *Transmembrane Protein 51* |  |
| *CCR7* | *Chemokine (C-C Motif) Receptor 7* | *TMEM88* | *Transmembrane protein 88* |  |
| *CDC6* | *Cell division cycle 6* | *TNS4* | *Aliases for TNS4 Gene* |  |
| *CDK10* | *Cyclin-Dependent Kinase 10* | *UBL4B* | *Ubiquitin-Like 4B* |  |
| *CELA2A* | *Chymotrypsin-Like Elastase Family, Member 2A* | *WC1.3* | *White Cap1.3* |  |
| *CTRC* | *Chymotrypsin C (caldecrin)* | *WIPF2* | *WAS/WASL Interacting Protein Family, Member 2* |  |
| *CYM* | *Chymosin* | *WNT5B* | *Wingless-Type MMTV Integration Site Family, Member 5B* |  |
| *DDX19B* | *ATP-dependent RNA helicase DDX19B* | *ZC3H11A* | *Zinc Finger CCCH-Type Containing 11A* |  |
| *EFHD2* | *EF-Hand Domain Family, Member D2* | *ZMAT3* | *Zinc finger, matrin-type 3* |  |
| *EXOSC6* | *Exosome Component 6* | *AJAP1* | *Adherens junctions associated protein 1* |  |
| *FANCA* | *Fanconi anemia, complementation group A* | *ALOX12B* | *Arachidonate 12-lipoxygenase, 12R type* |  |
| *FBXL14* | *F-Box And Leucine-Rich Repeat Protein 14* | *ALOX15B* | *Arachidonate 15-lipoxygenase type II* |  |
| *FBXL21* | *F-Box And Leucine-Rich Repeat Protein 21* | *ATOX1* | *Antioxidant Protein 1* |  |
| *G3BP1* | *Ras GTPase-activating protein-binding protein 1* | *CHMP1A* | *Charged Multivesicular Body Protein 1A* |  |
| *GLG1* | *Golgi Glycoprotein 1* | *CXCL14* | *Chemokine (C-X-C motif) ligand 14* |  |
| *GLRA1* | *Glycine receptor, alpha 1* | *DCC* | *Immunoglobulin Superfamily DCC Subclass Member 1* |  |
| *GUCY2D* | *Guanylate cyclase 2D, membrane (retina-specific)* | *DDX19A* | *DEAD (Asp-Glu-Ala-Asp) Box Polypeptide 19A* |  |
| *HOXC13* | *Homeobox C13* | *DNAH2* | *Dynein, axonemal, heavy chain 2* |  |
| *IGFBP4* | *Insulin-like growth factor-binding protein 4* | *DNAJC16* | *DnaJ (Hsp40) Homolog, Subfamily C, Member 16* |  |
| *IMPG1* | *Interphotoreceptor Matrix Proteoglycan 1* | *EBF1* | *Early B-Cell Factor 1* |  |
| *KRT222* | *Keratin 222, Type II* | *ERC1* | *ELKS/RAB6-Interacting/CAST Family Member 1* |  |
| *KBTBD1* | *Human Kelch-like protein 31* | *FMOD* | *Fibromodulin* |  |
| *KRT24-27* | *Keratin 24, Type I* | *FRMD4B* | *FERM Domain Containing 4B* |  |
| *LAMTOR* | *Late Endosomal/Lysosomal Adaptor, MAPK And MTOR Activator 1* | *GNG5* | *Guanine nucleotide-binding protein G(I)/G(S)/G(O) subunit Gamma-5* |  |
| *LAX1* | *Lymphocyte Transmembrane Adaptor 1* | *HOXC12* | *Homeobox C12* |  |
| *LECT2* | *Leukocyte cell-derived chemotaxin 2* | *HSPB9* | *Heat Shock Protein, Alpha-Crystallin-Related, B9* |  |
| *MC1R* | *melanocortin 1 receptor (alpha melanocyte stimulating hormone receptor)* | *KCNA2* | *Potassium Channel, Voltage Gated Shaker Related Subfamily A, Member 2* |  |
| *MRCL* | *Mannose receptor-like precursor* | *KCNB1* | *Potassium channel, voltage gated Shab related subfamily B, member 1* |  |
| *NDUFA12* | *NADH Dehydrogenase (Ubiquinone) 1 Alpha Subcomplex, 12* | *KCNMB3* | *Calcium-activated potassium channel subunit beta-3* |  |
| *OPTC* | *Opticin* | *KIAA1797* | *Human Uncharacterized protein* |  |
| *PDPR* | *Pyruvate Dehydrogenase Phosphatase Regulatory Subunit* | *MTPN* | *Myotrophin* |  |
| *PIK3CA* | *phosphatidylinositol-4,5-bisphosphate 3-kinase, catalytic subunit alpha.”* | *MYO6* | *Myosin VI* | |
| *PRELP* | *Proline/Arginine-Rich End Leucine-Rich Repeat Protein* | *NOS1AP* | *Nitric Oxide Synthase 1 (Neuronal) Adaptor Protein* |  |
| *PROK1* | *Prokineticin 1* | *NUCB2* | *Nucleobindin 2* |  |
| *PTGIS* | *Prostaglandin I2 (prostacyclin) synthase* | *OTX2* | *Orthodenticle homeobox 2.* |  |
| *RARA* | *Retinoic Acid Receptor, Alpha* | *OVOS2* | *Ovostatin 2* |  |
| *REN* | *Angiotensinogenase* | *PDPN* | *Podoplanin* |  |
| *RPF1* | *Ribosome production factor 1 homolog* | *PDPR* | *Pyruvate Dehydrogenase Phosphatase Regulatory subunit* |  |
| *RPS6KA2* | *Ribosomal Protein S6 Kinase, 90kDa, Polypeptide 2* | *PREP* | *Prolyl Endopeptidase* |  |
| *SEMA4A* | *Sema Domain, Immunoglobulin Domain (Ig), Transmembrane Domain (TM) and Short Cytoplasmic Domain, (Semaphorin) 4A* | *RBBP8* | *Retinoblastoma-binding protein 8* |  |
| *SLC16A4* | *Solute Carrier Family 16, Member 4* | *SCP2* | *Sterol Carrier Protein 2* |  |
| *SLC25A48* | *Solute Carrier Family 25, Member 48* | *SERPINA3-8* | *Serpin Peptidase Inhibitor, Clade A (Alpha-1 Antiproteinase, Antitrypsin* |  |
| *SLC22A17* | *Solute Carrier Family 22 (Organic Cation Transporter), Member 17 Peptide* | *SFT2D1* | *SFT2 Domain Containing 1* |  |
| *SMARCE1* | *SWI/SNF Related, Matrix Associated, Actin Dependent Regulator of Chromatin, Subfamily E, Member 1* | *WC1* | *White Cap1* |  |
| *SNRPE* | *Small Nuclear Ribonucleoprotein Polypeptide* | *ZNF280B* | *zinc finger protein 280B* |  |
| *BAF57* | *SMARCE1 (SWI/SNF related, matrix associated, actin dependent regulator of chromatin, subfamily e, member 1)* |  | |  |
